# CRISPR/Cas9-mediated genome editing induces gene knockdown by altering the pre-mRNA splicing in mice

**DOI:** 10.1186/s12896-018-0472-8

**Published:** 2018-10-03

**Authors:** Ji-Xin Tang, Dafeng Chen, Shou-Long Deng, Jian Li, Yuanyuan Li, Zheng Fu, Xiu-Xia Wang, Yan Zhang, Su-Ren Chen, Yi-Xun Liu

**Affiliations:** 1 State Key Laboratory of Stem Cell and Reproductive Biology, Institute of Zoology, Chinese Academy of Sciences, Beijing, 100101 China; 2 Affiliated Hospital of Guangdong Medical University, Zhanjiang, 524001 China; 3 State Key Laboratory of Integrated Management of Pest Insects and Rodents, Institute of Zoology, Chinese Academy of Sciences, Beijing, 100101 China; 4 University of Chinese Academy of Sciences, Beijing, 100049 China

## Abstract

**Background:**

Clustered Regularly Interspaced Short Palindromic Repeats/CRISPR associated protein 9 (CRISPR/Cas9) has been wildly used to generate gene knockout models through inducing indels causing frame-shift. However, there are few studies concerning the post-transcript effects caused by CRISPR-mediated genome editing.

**Results:**

In the present study, we showed that gene knockdown model also could be generated using CRISPR-mediated gene editing by disrupting the boundary of exon and intron in mice (C57BL/6 J). CRISPR induced indel at the boundary of exon and intron (5′ splice site) caused alternative splicing and produced multiple different mRNAs, most of these mRNAs introduced premature termination codon causing down expression of the gene.

**Conclusions:**

These results showed that alternative splicing mutants were able to generate through CRISPR-mediated genome editing by deleting the boundary of exon and intron causing disruption of 5′ splice site. Although alternative splicing was an unexpected outcome, this finding could be developed as a technology to generate gene knockdown models or to investigate pre-mRNA splicing.

**Electronic supplementary material:**

The online version of this article (10.1186/s12896-018-0472-8) contains supplementary material, which is available to authorized users.

## Background

CRISPR-mediated gene editing has been used in many organisms and transformed the study of gene editing [1–17]. Guide RNAs direct the Cas9 nuclease to the complementary target sites and at this site Cas9 nuclease cuts the double-strand DNA, generating a break in the genome. Repair of these double-strand DNA breaks can through the pathway of no homologous end-joining (NHEJ), which is able to introduce small insertions or deletions (indels). If the indels is not a multiple of three nucleotides shift, it is able to shift the reading frame and introduce premature termination codons (PTCs), which may result in mRNA degradation by nonsense-mediated decay (NMD) [18], thereby making the gene loss function. Over the past few years, CRISPR/Cas9 system has been proved to be a simple way to generate loss-of-function (LOF) mutations in the genome of many organisms, including mammals [1–17].

RNA polymerase II transcribes most eukaryotic genes, which can produce precursor messenger RNAs (pre-mRNA) that contain exons and introns. Pre-mRNA splicing involves the removal of introns and the joining of exons to form mature mRNA. In higher eukaryotes, pre-mRNA splicing plays an essential role in gene regulation. Through the alternative splicing (AS), which could inclusion of different exons in mRNA, one single gene can produce multiple different mRNAs. These mRNAs can be further translated into different proteins called splice variants. Through high-throughput sequencing technology, > 90% of human genes have been found undergo AS [19]. Moreover, genome-wide analyses have shown that about 95% the primary transcripts arriving from the multi-exon human genes undergo alternative pre-mRNA splicing [20]. Therefore, RNA splicing greatly increases the genomic complexity of higher eukaryotes. Although great efforts have been given, we are still partly known the mechanism of RNA splicing.

In the present study, we want to test the post-transcript effects caused by CRISPR-mediated genome editing in vivo of mammals. We found that CRISPR-mediated genome editing induces indel at the boundary exon 1 and intron 1 of mouse cyclin B3 (*Ccnb3*) disrupting 5′ splice site and causing alternative splicing to produce multiple different mRNA. Most of these mRNAs introduced PTCs resulting in down regulation of *Ccnb3* gene. These results show that alternative splicing mutation was able to generate using CRISPR/Cas9-mediated gene editing by deleting the boundary exon and intron disrupting the 5′ splice site. Previous study showed that CRISPR interference (CRISPRi) can efficiently repress the expression of targeted gene in *Escherichia coli* and mammalian cells, but it can not get a stable down regulation animal model [21]. In the present study, we showed a strategy to generate gene knockdown animal model using CRISPR/Cas9-mediated gene editing by generating alternative splicing mutants, although the strategy is not perfect, it is need more improvement to make it more predictable and controllable.

## Results

### Deletion the boundary exon 1 and intron 1 of *Ccnb3* using CRISPR/Cas9-mediated genome editing in mice

**Fig. 1:**
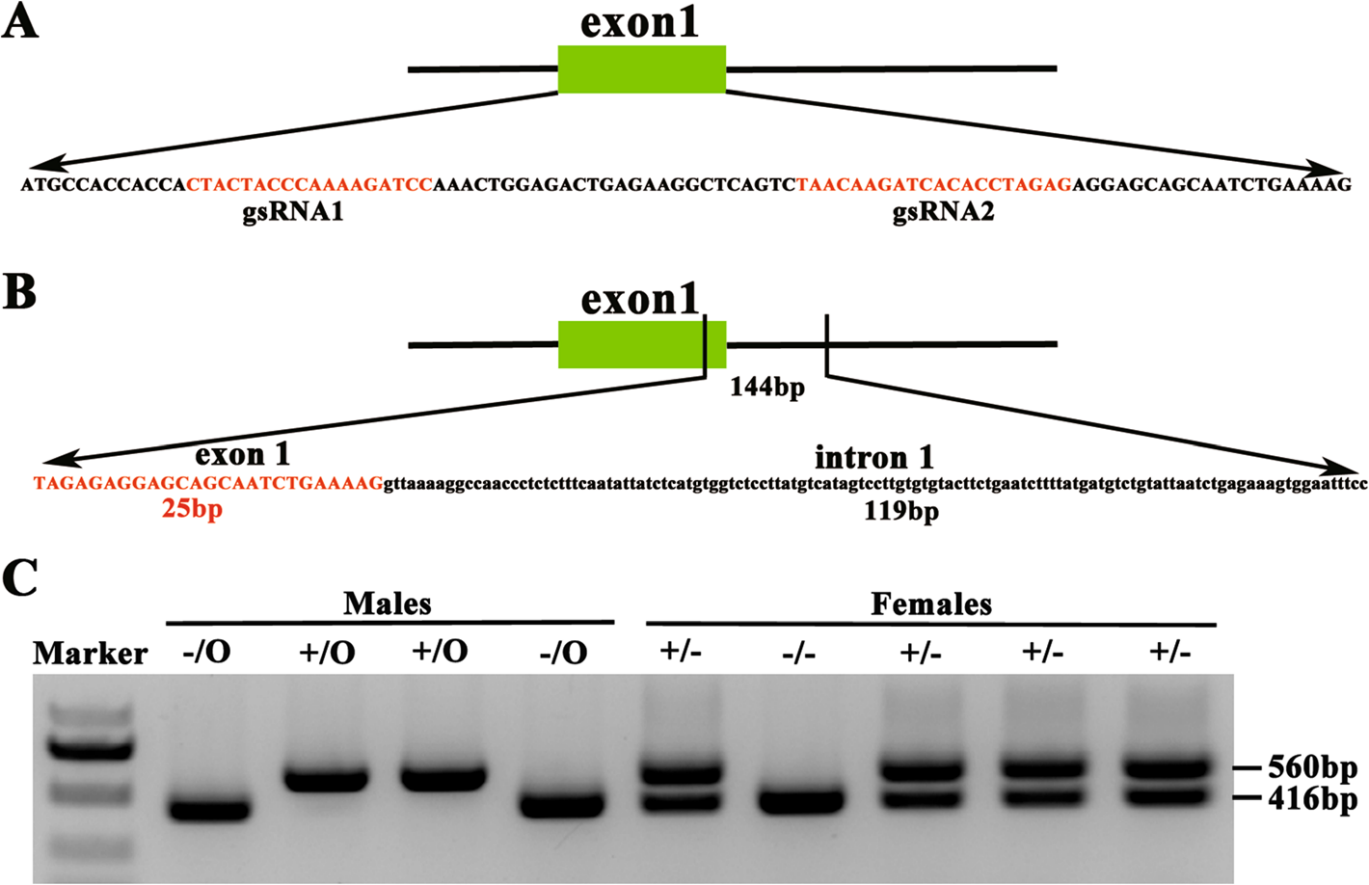
Deletion of the boundary of exon 1 and intron 1 of Ccnb3 by CRISPR/Cas9 system. **a** guide RNA binding site. sgRNA 1/2 specifically bind the exon 1 of Ccnb3. **b** The boundary of exon 1 and intron 1 of Ccnb3 (144 bp nucleotides) were deleted by CRISPR/Cas9 system. **c** Genotyping of mice. Every lane in Fig. 1c was from different mice. Ccnb3 wild type (+): 560 bp; Ccnb3 knockout (−: 144 bp was deleted): 416 bp

**Fig. 2:**
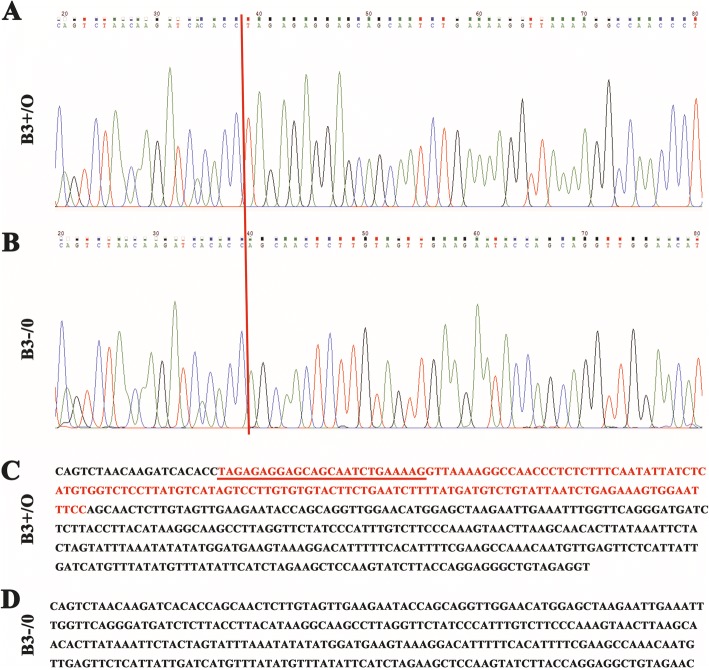
Verify the deletion of the boundary of exon 1 and intron 1 of Ccnb3 by DNA sequence. Part of Ccnb3’s exon 1 and intron 1 was amplified by polymerase chain reaction (PCR) and been sequenced. **a** The sequencing peak of part of Ccnb3’s exon 1 and intron 1 of Ccnb3+/O mouse. **b** The sequencing peak of part of Ccnb3’s exon 1 and intron 1 of Ccnb3-/O mouse. **c** The sequence of Ccnb3 exon 1/intron 1 boundary of Ccnb3+/O mouse. **d** The sequence of Ccnb3 exon 1/intron 1 boundary of Ccnb3-/O mouse. Red bar in **a** and **b** show the site that Ccnb3 sequence difference between Ccnb3+/O and Ccnb3-/O is appear. Red character in C is the lacking sequence in Ccnb3-/O. Among them, the underlined shows the deleted part of exon 1sequence of Ccnb3

Cas9 nickase mRNA and sgRNA 1/2 specifically targeting the exon 1 of *Ccnb3* were microinjected into the fertilized eggs of mice (Fig. 1a). Injected embryos were then cultured in vitro for one or 2 days before transplanted to the receptor pseudopregnancy female mice. The pup’s genotype had been identified by PCR and DNA sequence. We got one mutant mouse that missing 144 bp nucleotides at the boundary exon 1 and intron 1 of *Ccnb3* (Fig. 1b). PCR assay also show that the mutant mouse gets a 416 bp band lacking 144 bp nucleotides compared with wild type, which gets a 560 bp band (Fig. 1c). DNA sequence had proved that the missing 144 bp nucleotides located at the boundary exon 1 and intron 1 of *Ccnb3* (Fig. 2). These results indicate that we successfully disrupted the 5′ splice site by deleting the boundary exon 1 and intron 1 of *Ccnb3* using CRISPR/Cas9-mediated genome editing.

### Deletion of the boundary exon 1 and intron 1 of *Ccnb3* resulted in the alternative splicing of *Ccnb3* pre-mRNA

**Fig. 3:**
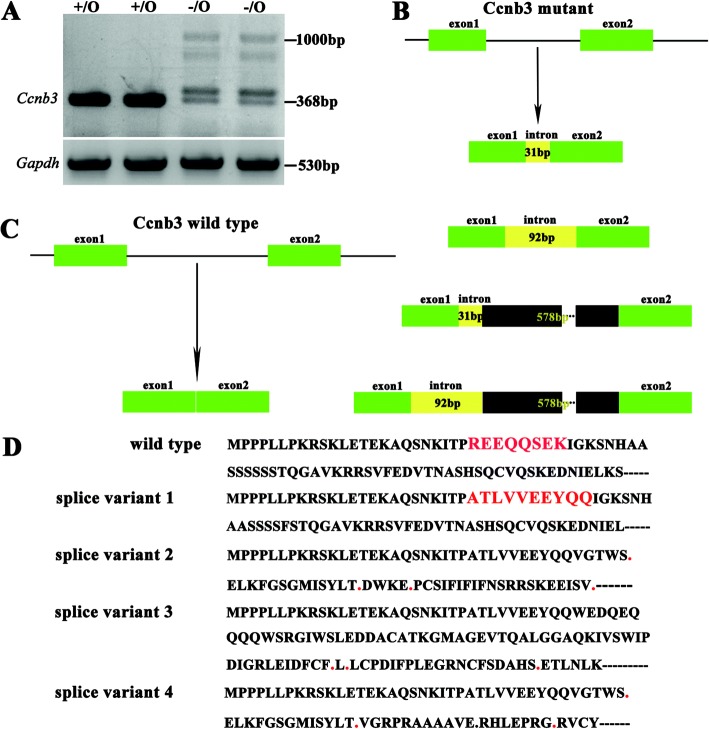
Knock down Ccnb3 expression by deletion its exon 1/intron 1 boundary using CRISPR/Cas9. **a** RT-PCR of Ccnb3 shows the expression of Ccnb3 in wild type (+/O) and mutant (−/O) mouse testes. **b** Ccnb3-/O mutant mouse’s Ccnb3 mRNA splicing. **c** Wild type mouse’s Ccnb3 mRNA splicing. **d** Wild type and four splicing mutation of Ccnb3’s part of amino acid sequence. .: stop codon. Red letter show the difference of amino acid sequence between wild type and splicing mutation 1 of Ccnb3

**Fig. 4:**
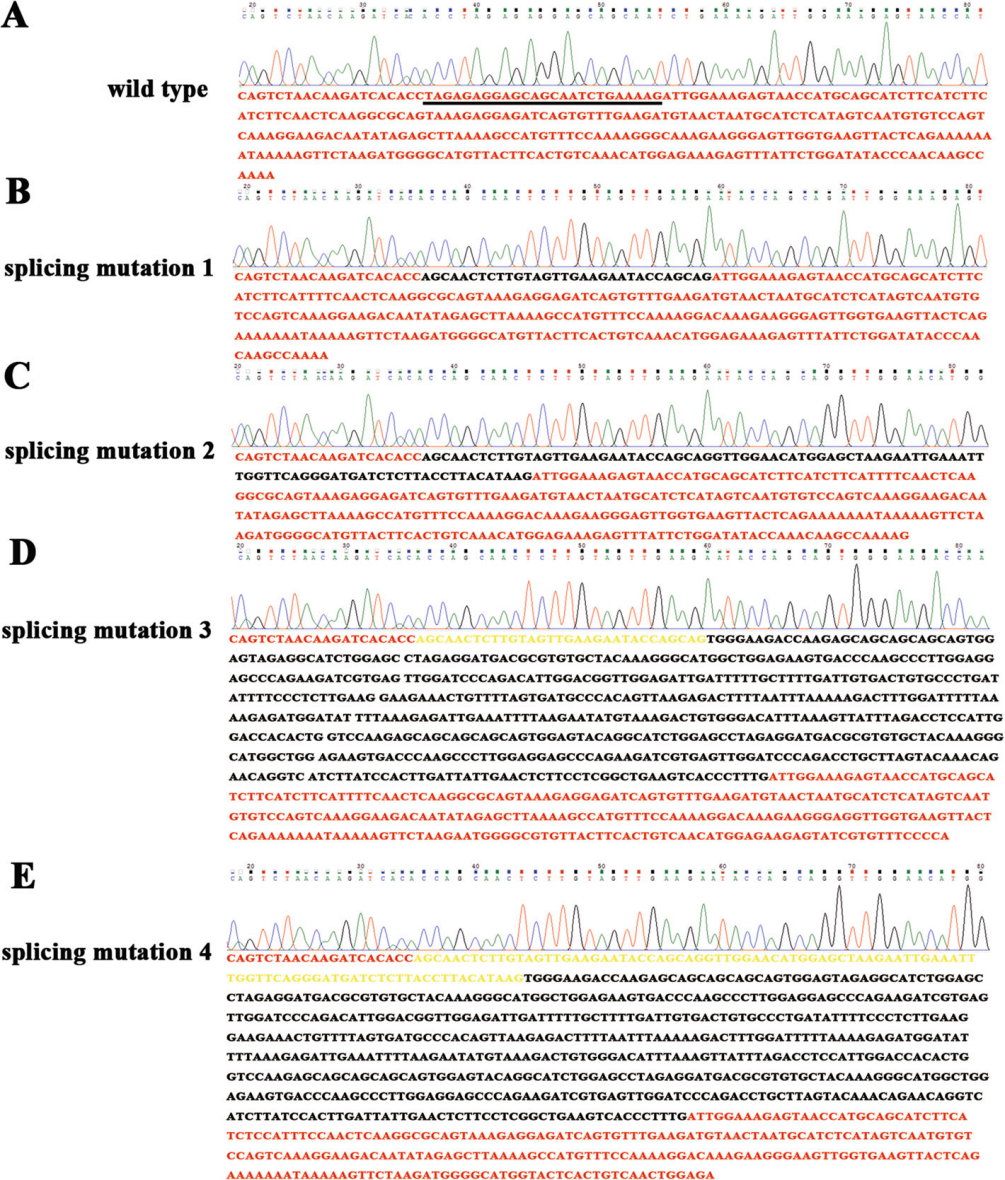
Deletion of exon 1/intron 1 boundary of Ccnb3 led to abnormal splicing and generated four splicing mutation. **a** Sequencing peak and sequence of Ccnb3 cDNA of wild type mouse. **b**, **c**, **d**, **e**. Sequencing peak and sequence of four splicing mutation of Ccnb3 cDNA of Ccnb3-/O mouse. **b** Splicing mutation 1: 25 bp exon 1 deletion and 31 bp intron 1 retention. **c** splicing mutation 2: 25 bp exon 1 deletion and 92 bp intron 1 retention. **d** splicing mutation 3: 25 bp exon 1 deletion and 31 bp intron 1 retention as well as extra 578 bp other nucleotides, 31 bp intron 1 retention was highlighted by yellow color. **e** splicing mutation 4: 25 bp exon 1 deletion and 92 bp intron 1 retention as well as 578 bp other nucleotides, 92 bp intron 1 retention was highlighted by yellow color

Considering the importance of the boundary exon and intron (5′ splice site) in pre-mRNA splicing, we propose that deletion of the boundary exon 1 and intron 1 of *Ccnb3* probably change the *Ccnb3* pre-mRNA splicing. Mammalian Ccnb3 is a prepachytene meiotic cyclins specifically expressed in germ cells [22], therefore we collected the total RNAs from the testes of control (+/o) and mutant (−/o) adult mice. Then, the total RNAs were reverse transcripted into cDNA and amplified the exon 1 to 3 by PCR. We found that the mutant mice have 4 bands when running the gel using the product of PCR products compared with one band in control mice (Fig. 3a). This result show that pre-mRNA alternative splicing probably happened in *Ccnb3* mutant mice. To verify this hypothesis we cloned the PCR products from control and mutant mice into pGEM-T vector. Then, we transfected DH5α *E. coli.* With these vectors. The transfected DH5α have been cultured in LB plate. When the clone grow, we using the clone to do the PCR. Then, the products of PCR have been sequenced. Compared with control, which have no alternative splicing, four splice variants were found in *Ccnb3* mutant mice (Fig. 3b, Fig. 3c, Fig. 4 and Additional file 1: Figure S1). In the first splice variant, 25 bp nucleotides of *Ccnb3* exon 1 are loss, 31 bp nucleotides of *Ccnb3* intron 1 are retention (Figs. 3b and 4b). This alteration not causes frame shift, albeit several nucleotides changing (Fig. 3d). In the second splice variant, 25 bp nucleotides of *Ccnb3* exon 1 are loss, 92 bp nucleotides of *Ccnb3* intron 1 are retention, causing *Ccnb3* frame shift (Figs. 3b and 4c). In the third splice variant, beyond the 25 bp *Ccnb3* exon 1 nucleotides loss and 31 bp intron 1 nucleotides retention, other 578 bp nucleotides that not belonging to intron 1 of Ccnb3 are included (Figs. 3b and 4d). In the fourth splice variant, beyond the 25 bp Ccnb3 exon 1 nucleotides loss and 92 bp intron 1 nucleotides retention, other 578 bp nucleotides that not belonging to intron 1 of Ccnb3 are in included (Figs. 3b and 4e). The unexpected 578 bp nucleotides found in third and fourth splice variant are the same sequence, it not belong to intron 1 of Ccnb3 (Additional file 1: Figure S1 and S2). The unexpected 578 bp nucleotides contain two copy of 128 bp nucleotides and these two copy of 128 bp nucleotides are the same sequence (Additional file 1: Figure S2). When we blasting the 578 bp nucleotides in mouse genome, we found that 353 bp or 352 bp nucleotides in the unexpected 578 bp nucleotides was found in mouse Chr1, Chr2, Chr3, Chr4, Chr5, Chr6, Chr7, Chr8, Chr9, Chr11, Chr13, Chr14, Chr15, Chr16, Chr17, Chr18 and ChrX (Additional file 1: Figure S2 and S3). Both the third and fourth splice variant are frame-shift variants and contain PTCs. In the wild type group, the pre-mRNA could splice normally (Figs. 3c, d and 4a); whereas in the mutant, four splice variant were found and three of them were frame-shift variants and contained PTCs (Fig. 3d).

### Knockdown of *Ccnb3* did not have effects on mouse spermatogenesis and male fertility

**Fig. 5:**
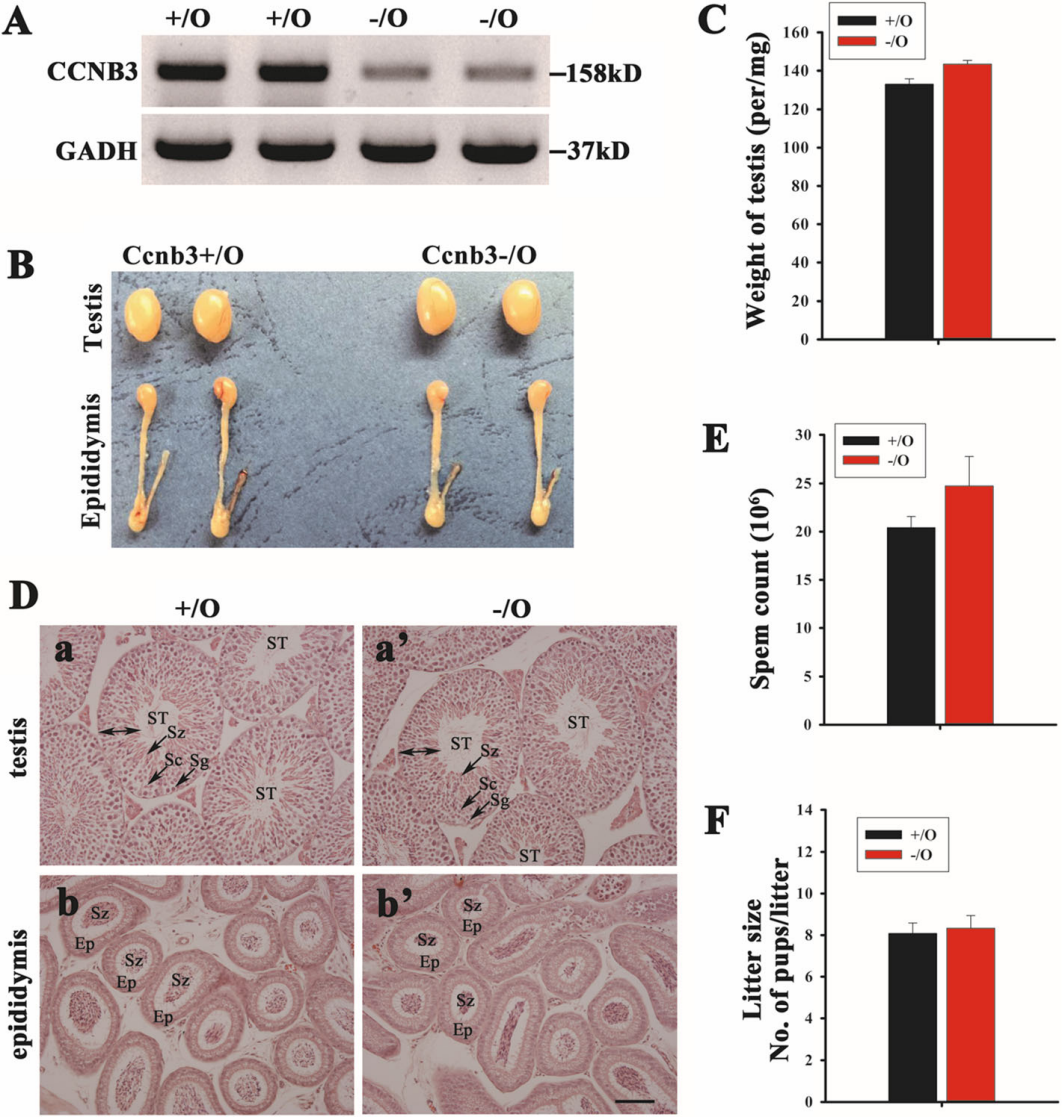
Knockdown Ccnb3 expression does not have an effect on spermatogenesis and male fertility. **a** Ccnb3 protein in adult wild type and Ccnb3 mutant mouse testes, detected by Western blot. **b** Adult wild type and Ccnb3 mutant mouse testes and epididymis. **c** Testis weight of adult wild type and Ccnb3 mutant mice. **d** H&E staining of adult testis and epididymis of wild type and Ccnb3 mutant. a and a’ were the cross section of testes; b and b’ were the cross section of epididymides. ST: seminiferous tubule; Sg: spermatogonia; Sc: spermatocytes; Sz: spermatozoa; ET: epididymal tubule. Arrows showed the Sg, Sc or Sz, respectively. Double-headed arrow showed the location of spermatogenic cells, which contains Sg, Sc and Sz. **e** Sperm count of adult wild type and Ccnb3 mutant mice. **f** Litter size of adult wild type and Ccnb3 mutant male mice. Bar = 100 μm

We found four splice variants in the mutant mice and only one was in-frame variant. Therefore, we propose that deletion of the boundary exon 1 and intron 1 of *Ccnb3* results in down-regulation of Ccnb3. To verify this hypothesis, we examined the protein of Ccnb3 in adult control and mutant mouse testes. Western blot assay showed that the protein of Ccnb3 was significant reduced in mutant mouse testes compared with control littermates (Fig. 5a). This result indicate that Ccnb3 protein level was indeed down regulated in the mutant mouse testes. Ccnb3 is not a ubiquitously expressed cyclin; it is specifically expressed in mammalian germ cells [22]. We then checked the effects of Ccnb3 down regulation in mouse spermatogenesis. We found that the testis and epididymis of control and mutant mice had not significant difference (Fig. 5b). The weight of testis is no significant difference between control and mutant mice (Fig. 5c). Histological assay of testis and epididymis showed that the spermatogenesis of control (+/O) and mutant (−/O) mice is no significant difference, both of them contain spermatogenic cells in seminiferous tubules and spermatozoa in epididymis (Fig. 5d). We then examined sperm count of control and mutant mouse epididymis and found that the sperm counts had no significant difference between control and mutant group (Fig. 5e). Furthermore, we tested the fertility of adult control and mutant male mice and found that they had the similar fertility (Fig. 5f). Collectively, these results suggest that down regulation of *Ccnb3* had no obviously effects on spermatogenesis and male fertility, indicating that *Ccnb3* probably redundant in mammalian spermatogenesis.

## Discussion

CRISPR/Cas9 system has been used in genome editing, generating the lose-function mutants in many organisms, including mammals [1–17]. In the present study, we showed here that mouse model of gene knockdown could be generated by CRISPR-mediated genome editing. By deleting the boundary exon 1 and intron 1 of *Ccnb3* causing alternative splicing and generating four splice variants, among these four splice variants, three of them are frame-shift variants resulting in the down regulation of *Ccnb3*. It is may be a good way to generate gene knockdown animal models to study the essential genes function in vivo as well as in vitro. However, we are not sure whether it is a common phenomenon or just an accident that deleting the boundary exon and intron can result in alternative splicing and gene knockdown. It need more work to prove that deleting exon and intron boundary to disrupting the 5′ splice site could indeed produce alternative splicing and gene knockdown.

Vertebrate Ccnb3 is a distantly related member of the B-type subfamily according to its sequence and properties [23]. However, it is has some differences compared to cyclin B1 (Ccnb1) and cyclin B2 (Ccnb2). *Ccnb3* has a huge exon, which can not be find in *Ccnb1* and *Ccnb2* [24]. Ccnb3 can associate with Cdk1 and Cdk2 [23], while Ccnb1 and Ccnb2 only associate with Cdk1 [25, 26]. Moreover, *Ccnb1* and *Ccnb2* is ubiquitously expressed in proliferating cells in many tissues [25, 26], while *Ccnb3* is specifically expressed in germ cells [22]. Although many efforts have been given, we are still unclear the function of *Ccnb3* in mammals. Previous study have shown that down-regulation of *Ccnb3* during the meiosis of spermatocytes is necessary for the normal meiosis of spermatocytes in mouse [27]. In the present study, we generated a *Ccnb3* knockdown mouse model by CRISPR/Cas9-mediated genome editing and found that *Ccnb3* knockdown mice were overtly normal and did not show any defects in spermatogenesis and male fertility. These results indicate that *Ccnb3* is probably redundant in mouse embryo development and spermatogenesis.

Moreover, we found that the alternative splicing is caused by selecting of different 5′ splicing site by spliceosome. Exon 1 and intron 1 of *Ccnb3* splice site is ‘aag/gtt’; 31 bp intron 1 retention splice site is ‘ag/gtt’; 92 bp intron 1 retention splice site is ‘aag/g’ (Additional file 1: Figure S4). The 31 bp and 92 bp intron 1 retention contain 4 nucleotides same to the exon 1 and intron 1 of *Ccnb3* 5′ splice site (Additional file 1: Figure S4). Different 5′ splicing site selection resulted in alternative splicing mutation and intron retention. We propose that spliceosome selects downstream similar splicing sites when the authentic 5′ splicing site had been deleted. This action resulted in the intron 1 of *Ccnb3* retention for 31 bp or 92 bp. Interestingly, another 578 bp nuclear acids appeared in the third and fourth splice variants besides the 31 bp and 92 bp intron retention. As *Ccnb3* gene locates in mouse X chromosome [24], we found that the 578 bp nucleotides in third and fourth splice variants is not belong to the intron 1 of *Ccnb3* (Additional file 1: FigureS1 and S2). We then blasted the 578 bp nucleotides in the mouse genome and found that 353 bp or 352 bp nucleotides of the 578 bp can be found in mouse Chr1, Chr2, Chr3, Chr4, Chr5, Chr6, Chr7, Chr8, Chr9, Chr11, Chr13, Chr14, Chr15, Chr16, Chr17, Chr18 and ChrX. We assume that the excrescent 578 bp nucleotides come from the mRNA *trans*-splicing, which occurs between two different RNA molecules and can create a chimeric molecule. [28, 29]. These observations indicate that deletion of boundary exon 1 and intron 1 of Ccnb3 is not only causing alternative selecting of 5′ splicing sites, but also resulting the *trans*-splicing of mRNA.

## Conclusions

In the present study, we investigated the post-transcript effects of CRISPR-mediated genome editing and showed that gene knockdown mouse model could generated by deleting the boundary of exon and intron to disrupt the 5′ splice site causing intron retention and alternative splicing. Moreover, we found that down regulation of *Ccnb3* did not have an effect on mouse embryo development, spermatogenesis and male fertility. Our study showed a new research direction of CRISPR-mediated genome editing, which could be used to study the mechanism of pre-mRNA splicing or to study gene function by generating gene knockdown animal models.

## Methods

### Animals

To get the mutant *Ccnb3* mice, the sgRNA 1 and 2 specifically tagerted the exon 1 of *Ccnb3* and Cas9 nickase mRNA were injected to the fertilized eggs with standard procedure. Mouse Genome DNA was collected from the 1 mm tail of mouse using lysis buffer (add 50 mM NaOH 180ul, boil in water for 40 min and then add 20ul 1 M Tris-Hcl pH 8.0, get 5ul as temple for the PCR). Genotypes were identified by PCR analysis using primers F1: 5’-GTGAGGTAGCTGAAGCCTAT-3′ and R1: 5’-GATAGAACCTAAGGCTTGCC -3′. The wild type groups got a band at 560 bp, while the mutant groups got a band at 416 bp. Mouse *Ccnb3* gene is locate in X chromosomes, therefore in male mice only one copy of *Ccnb3* and in female mice there are two copy of *Ccnb3*.

The mice used in this study, which mixed backgrounds of 129 and C57BL/6 J, were obtained from the Beijing Vital River Laboratory Animal Technology Co., Ltd., Beijing China. All animals were kept in accordance with the protocols approved by the guidelines of the Institutional Animal Care and Use Committee of the Institute of Zoology (IOZ), Chinese Academy of Sciences (CAS), Beijing, China. After the experimentation, the animals used in this study were handled by the Laboratory Animal Center of the Institute of Zoology (IOZ), Chinese Academy of Sciences (CAS) according to the guidelines of the Institutional Animal Care and Use Committee of the Institute of Zoology (IOZ), Chinese Academy of Sciences (CAS), Beijing, China. Our experiments in this study were adhered to the ARRIVE guidelines (Additional file 2).

### Total RNA isolation and RT-PCR analysis

Total RNA was extracted from adult testes using Trizol (TIANGEN, DP405–2, Beijing, China) according to the manufacturer’s protocol. Then, the total RNA concentration and purity were quantified using Nanodrop 2000 Spectrophotometer (Biolab, Scoresby, Vic., Australia). Before the reverse-transcribed of RNA, the genome DNA was cleared with the RNase-free DNase H. The RNA was then reverse-transcribed with 5× FastKing-RT SuperMix kit (TIANGEN, KR118–01, Beijing, China) according to the manufacturer’s protocols. We then use the *Gapdh* primers (For: 5′- ATGGTGAAGGTCGGTGTGAA-3’ Rev.: 5′- GCAGTGATGGCATGGACTGT-3′, 542 bp) and *Ccnb3* primers (F2: 5′- CCACCACCACTACTACCCAA-3′ R2: 5′- GGCTTGTTGGGTATATCCAG-3′, 368 bp product for wild type) to amplify *Gapdh* and *Ccnb3* and running the gel.

### Western blot analysis

Protein was extracted from the adult mouse testes and then separated on 10% SDS-PAGE gels. The protein in PAGE gels was transferred to PVDF membrane and then blocked with 5% nonfat milk (in 1X PBS). The membranes were then probed with primary antibodies CCNB3 (Invitrogen, PA5–37254, Rabbit, 1:1000, California, USA) or GAPDH (Bioworld, MB001, Mouse, 1:5000, Minnesota, USA) overnight. The membrane was washed with PBST for 3 times, 10 min per time. The membranes were then incubated with secondary antibodies conjugated to horseradish peroxidase (ZSGB-BIO, ZB-2301, Beijing, China; ZSGB-BIO, ZB-2305, Beijing, China) at a dilution of 1:5000 and detected by the ECL System (ThermoFisher Scientific, SuperSignal™ West Femto Maximum Sensitivity Substrate, 34096, Massachusetts, USA).

### Tissue collection and hematoxylin–eosin staining

Six Control or Ccnb3 mutant adult mouse testes and epididymis were collected and the testes were weighed, then fixed in 4% paraformaldehyde (PFA) for 48 h in 4 °C. The tissues were then embedded in paraffin. Five sections of each testis and epididymis (5 μm, taken 200 μm apart) were stained with hematoxylin–eosin (H&E) for normal histological analysis.

### Fertility testing

To test the fertility of the male mice, six 8-week-old control and six 8-week-old mutant mice were housed with wild-type proven fertility female C57 mice in a ratio of 1:2. Successful conception was defined by the presence of vaginal plug and subsequent visibly growing abdomen. The pregnant females were then separated and the litter sizes were recorded after birth.

### Statistical analysis

All experiments were performed at least in triplicate and the results were presented as mean ± SEM. Two groups were compared by using Student’s t-test and *P* < 0.05 (*) were considered significant.

## Additional files

Additional file 1: Intron 1 of *Ccnb3*, 578 bp other sequence and 5' splice site of 31 bp and 92 bp intron 1 retention splice mutants. (PDF 310 kb)

Additional file 2: The ARRIVE Guidelines. (DOC 37 kb)

## Abbreviations

AS: Alternative splicing
Ccnb1: Cyclin B1
Ccnb2: Cyclin B2
Ccnb3: Cyclin B3
CDK1: Cyclin dependent kinase 1
CDK2: Cyclin dependent kinase 2
CRISPR/Cas9: Clustered Regularly Interspaced Short Palindromic Repeats/ CRISPR associated protein 9
DSB: Double-strand breaks
Indels: Insertion-deletions
LOF: Loss-of-function
NHEJ: Non-homologous end joining
NMD: Nonsense-mediated mRNA decay
pre-mRNA: Precursor messenger RNAs
PTCs: Premature termination codons

## References

[1] Cong L, Ran FA, Cox D, Lin S, Barretto R, Habib N (2013). Multiplex genome engineering using CRISPR/Cas systems. Science.

[2] Mali P, Yang L, Esvelt KM, Aach J, Guell M, DiCarlo JE (2013). RNA-guided human genome engineering via Cas9. Science.

[3] Wang H, Yang H, Shivalila CS, Dawlaty MM, Cheng AW, Zhang F (2013). One-step generation of mice carrying mutations in multiple genes by CRISPR/Cas-mediated genome engineering. Cell.

[4] Li W, Teng F, Li T, Zhou Q (2013). Simultaneous generation and germline transmission of multiple gene mutations in rat using CRISPR-Cas systems. Nat Biotechnol.

[5] Yang D, Xu J, Zhu T, Fan J, Lai L, Zhang J (2014). Effective gene targeting in rabbits using RNA-guided Cas9 nucleases. J Mol Cell Biol.

[6] Nakayama T, Fish MB, Fisher M, Oomen-Hajagos J, Thomsen GH, Grainger RM (2013). Simple and efficient CRISPR/Cas9-mediated targeted mutagenesis in Xenopus tropicalis. Genesis.

[7] Hwang WY, Fu Y, Reyon D, Maeder ML, Tsai SQ, Sander JD (2013). Efficient genome editing in zebrafish using a CRISPR-Cas system. Nat Biotechnol.

[8] Yu Z, Ren M, Wang Z, Zhang B, Rong YS, Jiao R (2013). Highly effcient genome modifcations mediated by CRISPR/Cas9 in drosophila. Genetics.

[9] Wang Y, Li Z, Xu J, Zeng B, Ling L, You L (2013). The CRISPR/Cas system mediates efficient genome engineering in Bombyx mori. Cell Res.

[10] Friedland AE, Tzur YB, Esvelt KM, Colaiácovo MP, Church GM, Calarco JA (2013). Heritable genome editing in C. elegans via a CRISPR-Cas9 system. Nat Methods.

[11] Li Y, Zhang J, Chen D, Yang P, Jiang F, Wang X (2016). CRISPR/Cas9 in locusts: successful establishment of an olfactory deficiency line by targeting the mutagenesis of an odorant receptor co-receptor (Orco). Insect Biochem Mol Biol.

[12] Shan Q, Wang Y, Li J, Zhang Y, Chen K, Liang Z (2013). Targeted genome modification of crop plants using a CRISPR-Cas system. Nat Biotechnol.

[13] Jiang W, Zhou H, Bi H, Fromm M, Yang B, Weeks DP (2013). Demonstration of CRISPR/Cas9/sgRNA-mediated targeted gene modification in Arabidopsis, tobacco, sorghum and rice. Nucleic Acids Res.

[14] Sander JD, Joung JK (2014). CRISPR-Cas systems for editing, regulating and targeting genomes. Nat Biotechnol.

[15] DiCarlo JE, Norville JE, Mali P, Rios X, Aach J, Church GM (2013). Genome engineering in Saccharomyces cerevisiae using CRISPR-Cas systems. Nucleic Acids Res.

[16] Jiang W, Bikard D, Cox D, Zhang F, Marraffini LA (2013). RNA-guided editing of bacterial genomes using CRISPR-Cas systems. Nat Biotechnol.

[17] Tarasava Katia, Oh Eun Joong, Eckert Carrie A., Gill Ryan T. (2018). CRISPR-Enabled Tools for Engineering Microbial Genomes and Phenotypes. Biotechnology Journal.

[18] Popp MW, Maquat LE (2016). Leveraging rules of nonsense-mediated mRNA decay for genome engineering and personalized medicine. Cell.

[19] Wang ET, Sandberg R, Luo S, Khrebtukova I, Zhang L, Mayr C (2008). Alternative isoform regulation in human tissue transcriptomes. Nature.

[20] Pan Q, Shai O, Lee LJ, Frey BJ, Blencowe BJ (2008). Deep surveying of alternative splicing complexity in the human transcriptome by high-throughput sequencing. Nat Genet.

[21] Qi LS, Larson MH, Gilbert LA, Doudna JA, Weissman JS, Arkin AP (2013). Repurposing CRISPR as an RNA-guided platform for sequence specific control of gene expression. Cell.

[22] Nguyen TB, Manova K, Capodieci P, Lindon C, Bottega S, Wang XY (2002). Characterization and expression of mammalian cyclin b3, a prepachytene meiotic cyclin. J Biol Chem.

[23] Gallant P, Nigg EA (1994). Identification of a novel vertebrate cyclin: cyclin B3 shares properties with both A- and B-type cyclins. EMBO J.

[24] Lozano JC, Vergé V, Schatt P, Juengel JL, Peaucellier G (2012). Evolution of cyclin B3 shows an abrupt three-fold size increase, due to the extension of a single exon in placental mammals, allowing for new protein-protein interactions. Mol Biol Evol.

[25] Pines J, Hunter T (1991). Human Cyclins a and Bl are differentially located in the cell and undergo cell cycle-dependent nuclear transport. J Cell Biol.

[26] Draviam VM, Orrechia S, Lowe M, Pardi R, Pines J (2001). The localization of human Cyclins B1 and B2 determines CDK1 substrate specificity and neither enzyme requires MEK to disassemble the Golgi apparatus. J Cell Biol.

[27] Refik-Rogers J, Manova K, Koff A (2006). Misexpression of cyclin B3 leads to aberrant spermatogenesis. Cell Cycle.

[28] Lasda EL, Blumenthal T (2011). Trans-splicing. Wiley Interdiscip Rev RNA..

[29] Berger A, Maire S, Gaillard MC, Sahel JA, Hantraye P, Bemelmans AP (2016). mRNA trans-splicing in gene therapy for genetic diseases. Wiley Interdiscip Rev RNA.

