# Supplementary material for: CRISPR/Cas9-mediated genome editing induces gene knockdown by altering the pre-mRNA splicing in mice
Source: BMC Biotechnol. 2018 Oct 3;18:61. doi: 10.1186/s12896-018-0472-8 (PMC6171314; doi:10.1186/s12896-018-0472-8)
Supplement: Supplementary file 1 — Intron 1 of Ccnb3, 578 bp other sequence and 5' splice site of 31 bp and 92 bp intron 1 retention splice mutants. (PDF 310 kb) [file 12896_2018_472_MOESM1_ESM.pdf]

gttaaaaggccaaccctctctttcaatattatctcatgtggtctccttatgtcatagtccttgtgtgtact

tctgaatcttttatgatgtctgtattaatctgagaaagtggaatttcc**agcaactcttgtagttgaag**

aataccagcaggttggaacatggagctaagaattgaaatttggttcagggatgatctcttac

cttacataaggcaagccttaggttctatcccatttgtcttcccaaagtaacttaagcaacacttataa

attctactagtatttaaatatatatggatgaagtaaaggacatttttcacattttcgaagccaaacaatg

ttgagttctcattattgatcatgtttatatgtttatattcatctagaagctccaagtatcttaccaggagg

ctgtagtacattcttggtagtacgttttgtcaggaaaatattatcatattctgagtattcagattgagtat

ccctaatttgaaagttcaatatttaaaaatttcaaactctgaaatctttgagtgctgacctgattctgta

agtggaaaatgccatacttaatctcaagtaatggagtttacagtcaaactgcaggcatactaagag

ccaaagaggtcgctcagtggttaaagcagttgctgtacaagcatgcagaccagtttgaattccttg

catctgtgtaaaaggctgagtggacatggcagcttgcctgtaattctactgcaaaaggtataggca

aactctagactcaactcaactgagaaactggctctgaataagctgaggacaatagaagggcatct

ctgatgtcagccttgggcttccatatacacaggtatacattatcacatgcatatacttgcatacatata

acctacacatgtaaacatacatgcatatatacaacataataaaacagttccaggccactaaagttat

aaaattatataaaattgcgttcagactatgattttattatatatatatatatatatatatatatatatat

atatataatacaaaaggaattttggcagtaagggtggggagggtaatggatgagtaaatctatcaa

agtatttgatatagttgaacaaaatatcaatatgaagcccattgctttgaacagtgaatatgcccaag

tgaaattcgtttttaaaagaaaggctggacagatgggttggtggttaagaccttttgttgctcttagtg

aggattggagttcaattcccagcacccatatctggctggttacaacagcttgtagctccagctcca

gggtatctgaagccctcttctggcttctgtgggtacccacatttacatgtgcacacacatgctcattg

ttaaaatacattttggtgctggaaaaattgcttaggtttttttaaataatacttatctttaatctttttatag

agtccagtcattatccacctccaggtccaccctctgctatttcctcatttcattcctcctcctccctgtc

tccaagaggatgtcctcacccctccccctcctcactccctggggcctcaagtctcttgagggttag

ttaggtgcatcttctctcactgaggccagaccaggcagtcctctgctgtatatgtgtatatgctgtat

atgctgccttcttggtggctcattgtttgagaggtctctggggttcatgttagttgagactgctggtctt

catatggagtcaccctcctcctaagcctcttccagcctttccctaatcaacaacagcacccccccc

cccggcttctgtccattggttgggtgtaagtatctgtgtctgactctttcagctgcttgttgggcttctc

agaggacaggcatgtctgtgagcacactataacatcagtaatagtgtcaggccttggagcctcctt

tccctcagactcttccatttttgtttcttcagttctttcagacaggaacaattctgggtcagagtttttac

tgtgggatgccatccccatccctccacttgatgccctgtctttctcctggaagtggactctactgcttt

tccagagaatctaggttaaatttccagcacctacattgagaagttcacaagtaccttaactccagttc

caggggggattcccatgcctgcaatcatatccacagaccaaaacatagataatacatacacataatt

aaaaatagtaaaaatacatctttcaaaaagaataattaaaaagaaaaaatcctgggtcccatcctga

tgatatttaattatgtatatataaatacttcaaatgagtagaaggtgaaatccaagaaacttctagtcc

caaacatgttagctataggttacttaaccagtattagattttaagaatgtatcggtgcacttaaacttg

cctgatttgactttggagtagtttaacactgattgaccttttcatcctggaatattctatgttcattacca

aatatattttaacaatagttatattaacacttatgaatcatcatcagggagaatttcctgaaggagaga

gcattgggccaatataatttttttgatagtgctggacatgtagcttagtggcagaggcgcttgcctatc

aggtccaaggtcctgtgttcaatctctagcactgcagactttttttatgtgccgataaacattttcttttttt

gtttgttttttttgggttttgttttgttttgttttgttttgttttgtttttcgagacagggtttctctgcatagccct

ggctgtcctggaactcactttgtagaccaggctggcctcgaactcagaaatcctcctgcctctgcc

tcccaagtgctgggattaaaggcgtgagccaccacgcccggcttcaataaacattttcaattattttt

tactttgttgtagtaaaatgattttcatggagggaatgagacattttcccatccaaatttatcaagaaa

gatttcatacctaattgagcttttgtttatatcaaatgttctgactgtagttacgatattgtaagtttacttt

catggctattggttttccagttcaaccttattttggacatattttgtaaatataagtttttttattattacgt

attttcctcaattacatttccaatgctatcccaaaagtcccccacaacctccccccactcccctacc

cacccattcccatttttggccctggcgttcccctgtactggggcatataaagtttgcctgtacaatg

ggcctctctttccagtgatggccgactaggccatctttttgatacatatgcagctagagtcaagagc

tctagggtactagttagttcataatgttgcacctacagggttgcagatccctttagctccttgggtact

ttctctagctcctccattgggggccctgtgatccatccaatagttgactgtgagcatccacttctatgt

ttgctaggccccagcctagtctcacaagagacagctatatcacggtccttgcaacaaacgcttgct

agtgtatgcaatggtgtcatcgtttggaagctagttatgggatggatccctggatatggcagtctct

agatggtccaacctttggtctcagctccaaactttgtctctgtaactccttccatgggtgataatataa

gttttattaagtcctgtttctactgttaatttgaaattgaggagattattttaacagtaataccaagtgag

aattagaagactaagtgtttatttttttagcctatttagaagaaaagtgatgttttatcatttgctaattattt

ctattaggattatttattgcttattatttataagataaaaatgaatagggtttttaaagctgaaatagaat

gggaaataattcttagaattgctcctttttttggaatggtgtattacatcatgatacattctccaagtttt

ggagcatttactgtgtcatatactgttaatgatgtagttagcacaaaactagtgtcatatccaagtgtt

ctgtttaataattgtatgaactttcttatatagataaggcagagtttgttcttagaattggaaaagagaa

atctgataattaatagatagatattcaccaaatagtaactgttaaaagatagaaaaatgccacaaga

gtgatttctcctaacttccaacttcatagagatagtcttagtcatgtgggagtaggttaaggcatgtat

agtaccaagaaagaaaaacttcgttgcttcctatatggtttcatttttccttgatcacttattttctctgc

catgagtctcacatatcaaataacattagagataatctgtattaactattttcttaaaataattttttcat

gtgatgtttacaagtattcagtactacatagctttaaagtactttttgcatttaaaatttatatagattga

aaaaatatatattctatagtagaaacattgcttaggctatgcaagactctgggtccaatctttagtgca

attacaataaaacacttttaaaagtatattttcttttgtaatccataaagactctgtaaggtaggttatca

ttccaattttactgatgagaaaattagagtctcatacgttaagtgactcttccaaggtcaatatttttaa

aaaaaattgagctgaatctggactagtacctaggtttgtccctattgaatatgtattaaagttcactta

gtataagaaagacttaattacttgtgctttttaactaacttagttggaggtatttctatagaaagactca

ctactccttatgatgatagttaatcctggatagtataaccataactaggagaagaagagaataaatc

agtttcaccaaacttactcattttgaaatttttactgaagaccatgtaatctcttcatgtccctattatca

atgactatattcttcattgactgttaaagatctctgaatactaaagctttttactgtagttcataggcttta

ttatacatatcccagttacttggacatggtagagtctatccacaccctgaatgtgacccaaagttcat

aaaggggctatttataattgtccatgttcatctgattcttatattttatag

Figure S1. The entire intron 1 sequences of Ccnb3. Sequences corresponding to the 31 bp intron 1 retention was underlined and the 92 bp intron 1 retention was highlighted in a yellow color.

TGGGAAGACCAAGAGCAGCAGCAGCAGTGGAGTAGAGGCATCTGGAGCC
TAGAGGATGACGCGTGTGCTACAAAGGGCATGGCTGGAGAAGTGACCCA
AGCCCTTGGAGGAGCCCAGAAGATCGTGAGTTGGATCCCAGACATTGGAC
GGTTGGAGATTGATTTTTGCTTTTGATTGTGACTGTGCCCTGATATTTTCCC
TCTTGAAGGAAGAAACTGTTTTAGTGATGCCCACAGTTAAGAGACTTTTA
ATTTAAAAAGACTTTGGATTTTTAAAAGAGATGGATATTTTAAAGAGATT
GAAATTTTAAGAATATGTAAAGACTGTGGGACATTTAAAGTTATTTAGAC
CT(C)CATTGGACCACACTGGTCCAAGAGCAGCAGCAGCAGTGGAGTACAG
GCATCTGGAGCCTAGAGGATGACGCGTGTGCTACAAAGGGCATGGCTGGA
GAAGTGACCCAAGCCCTTGGAGGAGCCCAGAAGATCGTGAGTTGGATCCC
AGACCTGCTTAGTACAAACAGAACAGGTCATCTTATCCACTTGATTATTGA
ACTCTTCCTCGGCTGAAGTCACCCTTTG

Figure S2. The inserted 578bp nucleotides in the third and fourth alternative splicing mutants. Red characters show the two copy of 128bp nucleotides. The high light character with yellow show the 353bp (contain the last ‘C’) or 352bp (no contain the last ‘C’) nucleotides which can be find in mouse Chr1, Chr2, Chr3, Chr4, Chr5, Chr6, Chr7, Chr8, Chr9, Chr11, Chr13, Chr14, Chr15, Chr16, Chr17, Chr18 and ChrX.

ACTIONS	QUERY	SCORE	START	END	QSIZE	IDENTITY	CHRO	STRAND	START	END	SPAN
browser details	YourSeq	352	1	353	578	100.0%	1	-	141058322	141058935	614
browser details	YourSeq	351	1	352	578	100.0%	X	-	130648046	130648659	614
browser details	YourSeq	351	1	352	578	100.0%	9	-	29740287	29740899	613
browser details	YourSeq	351	1	352	578	100.0%	9	-	17261884	17262496	613
browser details	YourSeq	351	1	352	578	100.0%	8	-	44783673	44784285	613
browser details	YourSeq	351	1	352	578	100.0%	7	-	128039543	128040155	613
browser details	YourSeq	351	1	352	578	100.0%	5	-	72345194	72345806	613
browser details	YourSeq	351	1	352	578	100.0%	5	-	69503761	69504373	613
browser details	YourSeq	351	1	352	578	100.0%	5	-	59573025	59573638	614
browser details	YourSeq	351	1	352	578	100.0%	5	-	18973525	18974137	613
browser details	YourSeq	351	1	352	578	100.0%	3	-	69229096	69229708	613
browser details	YourSeq	351	1	352	578	100.0%	2	-	165477878	165478490	613
browser details	YourSeq	351	1	352	578	100.0%	18	-	57560966	57561578	613
browser details	YourSeq	351	1	352	578	100.0%	17	-	83153546	83154158	613
browser details	YourSeq	351	1	352	578	100.0%	17	-	73319112	73319724	613
browser details	YourSeq	351	1	352	578	100.0%	13	-	14707881	14708493	613
browser details	YourSeq	351	1	352	578	100.0%	11	-	121612406	121613018	613
browser details	YourSeq	351	1	352	578	100.0%	11	-	64457299	64457911	613
browser details	YourSeq	351	1	352	578	100.0%	11	-	43237806	43238418	613
browser details	YourSeq	351	1	352	578	100.0%	X	+	17476419	17477031	613
browser details	YourSeq	351	1	352	578	100.0%	8	+	95912608	95913221	614
browser details	YourSeq	351	1	352	578	100.0%	4	+	78478007	78478619	613
browser details	YourSeq	351	1	352	578	100.0%	4	+	21093863	21094475	613
browser details	YourSeq	351	1	352	578	100.0%	3	+	27318686	27319299	614
browser details	YourSeq	351	1	352	578	100.0%	2	+	7657188	7657800	613
browser details	YourSeq	351	1	352	578	100.0%	16	+	81780865	81781477	613
browser details	YourSeq	351	1	352	578	100.0%	14	+	114822848	114823460	613
browser details	YourSeq	351	1	352	578	100.0%	13	+	44489007	44489619	613
browser details	YourSeq	350	1	352	578	100.0%	13	+	62314151	62314764	614
browser details	YourSeq	349	1	352	578	99.8%	X	-	38849827	38850439	613
browser details	YourSeq	349	1	352	578	99.8%	15	-	24897029	24897642	614
browser details	YourSeq	349	1	352	578	99.8%	14	-	29001727	29002340	614
browser details	YourSeq	349	1	352	578	99.8%	11	-	52553975	52554587	613
browser details	YourSeq	349	1	352	578	99.8%	8	+	98940331	98940944	614
browser details	YourSeq	349	1	352	578	99.8%	7	+	35933816	35934429	614
browser details	YourSeq	349	1	352	578	99.8%	7	+	28658353	28658965	613
browser details	YourSeq	349	1	352	578	99.8%	6	+	53465013	53465625	613
browser details	YourSeq	349	1	352	578	99.8%	2	+	129612492	129613105	614
browser details	YourSeq	349	1	352	578	99.8%	14	+	75799243	75799856	614
browser details	YourSeq	349	1	352	578	99.8%	14	+	60960867	60961479	613
browser details	YourSeq	349	1	352	578	99.8%	11	+	91048131	91048743	613
browser details	YourSeq	349	1	352	578	99.8%	1	+	150271005	150271617	613

Figure S3. Blast 558bp nucleotides in the mouse genome (http://genome.ucsc.edu/cgi-bin/hgBlat). 353bp or 352bp nucleotides of the 577bp unexpected nucleotides could be found in mouse Chr1, Chr2, Chr3, Chr4, Chr5, Chr6, Chr7, Chr8, Chr9, Chr11, Chr13, Chr14, Chr15, Chr16, Chr17, Chr 18 and ChrX.

atgccaccaccactactacccaaaagatccaaactggagactgagaaggctcagtctaacaaga

tcacacctagagaggagcagcaatctgaaaag/gttaaaaggccaaccctctctttcaatattatct

catgtggtctccttatgtcatagtccttgtgtgtacttctgaatcttttatgatgtctgtattaatctgaga

aagtggaatttccagcaactcttgtagttgaagaataccagcag/gttggaacatggagctaagaa

ttgaaatttggttcagggatgatctcttaccttacataag/gcaagccttaggttctatcccatttgtctt

cccaaagtaactta

Figure S4. Exon 1 and partial intron 1 of Ccnb3. Green characters show the exon 1 of Ccnb3 and black characters show the partial intron 1 of Ccnb3. Underline characters show the deleted nucleotides in Ccnb3 mutant. /: indicates the splice sites in Ccnb3 wild type and mutant. High light characters (yellow) show the conserved sequences in Ccnb3 wild type and mutant splice sites. aag/gtt: exon 1 and intron 1 of Ccnb3 splice site. ag/gtt: 31 bp intron 1 retention splice site. aag/g: 92bp intron 1 retention splice site.
